# Response to “Early prediction of noninvasive ventilation failure in COPD patients: derivation, internal validation, and external validation of a simple risk score”

**DOI:** 10.1186/s13613-019-0614-8

**Published:** 2019-12-18

**Authors:** Jun Duan, Linfu Bai

**Affiliations:** Department of Respiratory and Critical Care Medicine, The First Affiliated Hospital of Chongqing Medical University, Youyi Road 1, Yuzhong District, Chongqing, 400016 People’s Republic of China

Dear editor,

We appreciate the letter by Ennouri et al. [1] and have read it with great interest. Five variables were used to develop the risk score (heart rate, acidosis, consciousness, oxygenation, and respiratory rate [HACOR]) to predict NIV failure in patients with chronic obstructive pulmonary disease (COPD) [2]. These variables were classified into clinically meaningful categories. We did not use one cutoff for each element to develop this risk score as some important information may be omitted. For example, if the 7.35 is a cutoff value for pH, the risk for NIV failure was the same in patients with pH of 7.30 and 7.10. Obviously, it was largely different between the two patients in real word. In addition, many risk scores (e.g., APACHE II) also used multiple cutoff variables to indicate different risk. Therefore, we believe multiple cutoff variables are better than one.

Taking into account the HACOR score variability from initiation to the other, the area under the receiver operating characteristic curves (AUC) was 0.85 for HACOR score at initiation and 0.74 for variability from initiation to 2 h of NIV (Fig. 1). It means the variability is less accurate than the actual value to predict NIV failure. Similar outcomes were confirmed at 12 and 24 h of NIV. These results tell us that the actual value is better than the variability to predict NIV failure. For example, if the HACOR score is 2 points at initiation of NIV, the risk for NIV failure is low. After 2 h of NIV, when it increases to 4 points, the absolute risk for NIV failure is still low. However, even the score decreases from 15 at initiation to 10 at 2 h of NIV, and the risk for NIV failure remains high. Therefore, we believe the actual value may be more accurate to predict NIV failure than variability.

**Fig. 1:**
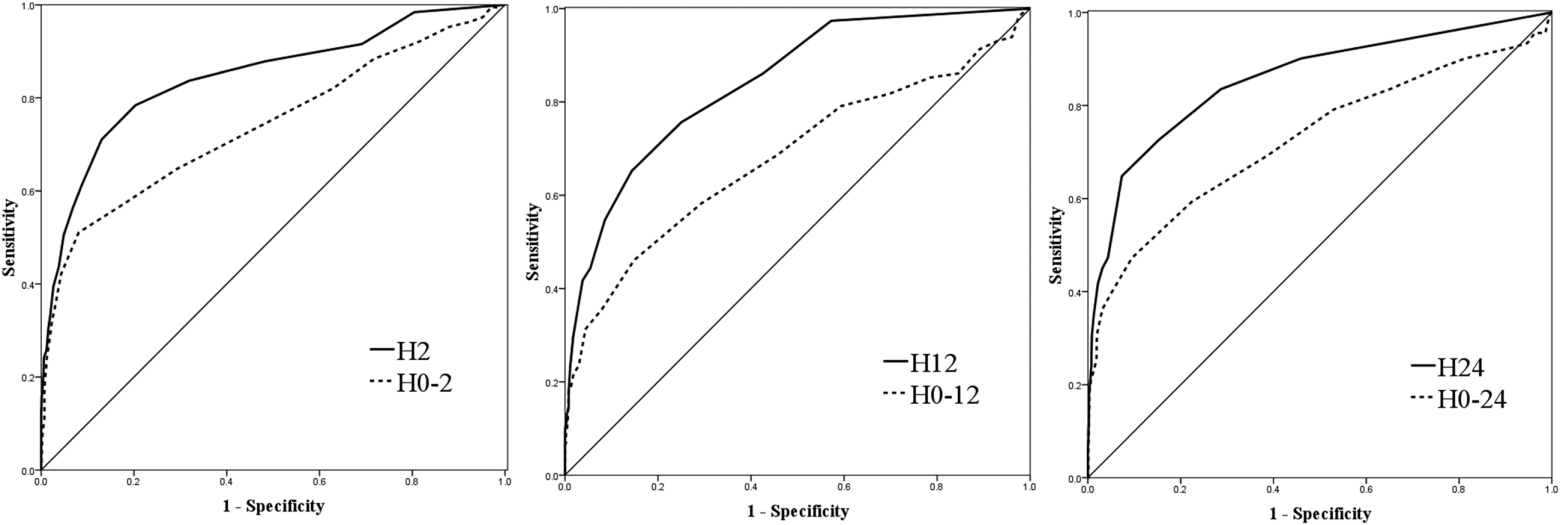
The AUC for prediction of NIV failure. H2, H12, and H24 mean the HACOR score collected at 2, 12, and 24 h of NIV. H0–2, H0–12, and H0–24 mean the difference from initiation to 2, 12, and 24 h of NIV, respectively

## Data Availability

The datasets analyzed during the current study are available from the corresponding author on reasonable request.

## Abbreviations

NIV: noninvasive ventilation
COPD: chronic obstructive pulmonary disease
AUC: area under the curve of receiver operating characteristics
HACOR: heart rate, acidosis, consciousness, oxygenation, and respiratory rate
